# Prophylactic Chemotherapy with Methotrexate Leucovorin in High-Risk Hydatidiform Mole

**DOI:** 10.31557/APJCP.2020.21.6.1755

**Published:** 2020-06

**Authors:** Soheila Aminimoghaddam, Fatemeh Mahmoudzadeh, Marzieh Mohammadi

**Affiliations:** 1 *Department of Gynecology and Oncology, Iran University of Medical Sciences, Tehran, Iran. *; 2 *Department of Emergency Medicine, Tehran University of Medical Sciences, Tehran, Iran. *

**Keywords:** Gestational trophoblastic neoplasia, high-risk mole, prophylactic chemotherapy

## Abstract

**Aim:**

Gestational Trophoblastic Neoplasia (GTN) is used to describe a group of malignant gestational tumors originating from the placenta. The chance of having malignant GTN is high in a high-risk molar pregnancy. The main aim of this study is to investigate the effectiveness of using prophylactic chemotherapy in high-risk molar pregnancy to prevent malignant GTN.

**Method:**

In this case-control retrospective study, all patients with high-risk mole referred to Firoozgar and Akbarabadi Hospitals affiliated with Iran University of Medical Sciences (IUMS) from 2003 to 2013 were divided into two groups of recipient and non-recipient of methotrexate prophylactic chemotherapy.Demographic information including age, parity, weight, serum βHCG before and after the intervention, level of liver function tests (LFT) and GTN were analyzed.

**Results:**

There were 102 patients with a mean age of 27.13 years (SD= 0.37), and 51 patients (50 %) received prophylactic Methotrexate (MTX), and others were the non-receivers. Finally, 23 patients (22.5%) were inflicted with GTN, and 79 (77.5 %) did not. The average time of βHCG spontaneous remission between the groups were 2.5 (SD=1.33) and 3.2 (SD=1.21), for the recipient and non-recipient, respectively, which showed a significant difference (p).

**Conclusion:**

This study concludes that prophylactic chemotherapy with MTX and leucovorin may be capable of reducing GTN, which supports the prescription of MTX in high-risk mole, especially in countries with limited resources. The toxicity of methotrexate can be reduced with the addition of leucovorin.

## Introduction

Gestational Trophoblastic disease is a continuum, which covers a wide range of states from benign molar pregnancy to Gestational Trophoblastic Neoplasia (GTN) (Aminimoghaddam et al., 2018; Rachdi et al., 2019). Molar pregnancy is divided into three categories; complete, partial, and invasive mole, and malignant form (Hurteau, 2003). Malignant form is a rare disease, which is divided into three categories; choriocarcinoma, Placental Site Trophoblastic Tumor (PSTT), Epithelioid Trophoblastic Tumor (Aminimoghaddam et al., 2016). 

All three types of benign molar pregnancy can be detected using histopathology, karyotype, and gross appearance of placenta. Hysterectomy should be used to detect invasive mole (Aminimoghaddam and Maghsoudnia, 2017). To define the characteristics of each type of molar pregnancies, trophoblastic proliferation should be addressed. In a complete mole, trophoblastic proliferation is excessive, while, a few trophoblastic tissue is detected in partial mole (Seckl et al., 2010). In invasive mole, trophoblastic invasion into the myometrium followed by villous formation are detected (Milani, 2017). Choriocarcinoma is the most malignant form of GTN, which has a high tendency to develop metastasis (Berkowitz and Goldstein, 1996). Pathology report shows bleeding and necrosis without villous formation in placental tissue. The GTN can be detected in different situations; after evacuation of molar pregnancy (with 50% occurrence rate), after term pregnancy (with 25% occurrence rate), and after ectopic pregnancy abortion (with 25% occurrence rate) (Altieri et al., 2003). 

Regarding predisposing factors, old women (who are in advanced ages) or women who had previous molar pregnancies are highly susceptive to have complete molar pregnancy (Altieri et al., 2003). High-risk mole, which originated mainly from complete moles, is the most important factor that causes GTN. High-risk mole is diagnosed if any of the following conditions meet; if ΒHCG is more than or equal to 100,000, if theca lutein cyst size is bigger than or equal to 6 cm, if age is more than or equal to 40 years, and if uterine size is larger than expected gestational age (Aminimoghaddam et al., 2018; Berkowitz and Goldstein, 2009). 

Regarding diagnosing of GTN, literature shows that it can be diagnosed if any of the following conditions meet: if βHCG is rises for 3 successive weeks after evacuation of molar pregnancy, if ΒHCG decreases less than 10% after 4 weeks of molar pregnancy evacuation (plateau), if choriocarcinoma had been detected in histology, if ΒHCG level can be measured after 6 months of evacuation of molar pregnancy (Schorge et al., 2000). Diagnosis is followed by anatomical staging (recommended based on FIGO (Oncology, 2009)) and scoring (recommended by WHO (Seckl et al., 2010)), which paves the way of treatment with single agent or multiple agents’ chemotherapy (Aminimoghaddam et al., 2018; Yarandi et al., 2016).

The GTN can be reduced by taking methotrexate or actinomycin-D, as the prophylactic treatments, before or within the suction curettage process. Actinomycin –D is generally used in bolus doses, while MTX can be used in single dose or in combination with leucovorin. These two drugs are generally used in observational studies. 

In summary, molar pregnancy is prevalent in the middle eastern countries (Gül et al., 1997). This proliferation, which engenders in having multiple patients with GTN, enables the authors to investigate the effect of using MTX- leucovorin as the prophylactic drugs. Moreover, this study addresses the toxicity of using methotrexate. 

## Materials and Methods

The present analytical, case-control retrospective study was performed in patients with high-risk moles referred to Akbarabadi and Firoozgar teaching hospitals, affiliated to Iran University of Medical Sciences (IUMS) in Tehran, during 2003-2013. 102 female patients were included in this study, which had at least one of the following inclusion criteria (Wolfberg et al., 2006). 

1. Uterine size larger than the gestation age

2. βHCG more than 100,000 (IU/ml)

3. Ovarian theca lutein cyst larger than 6cm

4. Age > 40 years

It should be noted that by having only one of these conditions, the patient is counted as a high-risk mole patient. Five groups of patients were not included in this study, which were:

1. Poor physical conditions

2. Abnormal liver function test

3. Abnormal CBC and renal function test

4. Lack of self-interest to take prophylactic chemotherapy

5. Incomplete records

Study protocol was approved by the Institutional Review Board (IRB) at Iran University of Medical Sciences (approval number: IR.IUMS.FMD.REC 1393.1536). In this study, all the principles of the Declaration of Helsinki (ethics in medical research on humans) and Code of Ethics adopted by the Ministry of Health at all stages of Association were followed. The data was collected from patients records and no extra expenses were imposed on the patients (Ghaoomeh et al., 2016).

The patients were divided into two groups, randomly: Group I) those receiving prophylactic chemotherapy (Methotrexate 1mg/kg in the odd days (1,3,5,7) and leucovorin 0.1 mg/kg (IM) in the even days (2,4,6,8); and Group II) those who did not receive it. Patients’ demographic data included age, number of pregnancies, weight, vaginal bleeding duration, improvement period, βHCG serum level before and after intervention, liver function tests levels, and finally diagnosis of GTN were recorded. The data were entered into a previously designed checklist. 

GTN was defined considering the presence of one of the following cases (Aminimoghaddam et al., 2018; Seckl et al., 2010):

1. Increase in the βHCG to 10% in three consecutive periods

2. Reduction of βHCG to 10% following four times measurement (Plateau)

3. Evidence of choriocarcinoma in the pathology

4. Having a high βHCG level in 6 months after suction curettage.

We measured the liver function test level four times per cycle, and we measured it at the same day that the patient received the methotrexate. In females with abnormal liver function tests (two times of the liver enzyme), the drug use was discontinued, and was resumed following normalization of the patient’s laboratory data. We followed them up for a year by taking βHCG level each month.

*Statistical analysis*

The data were collected and the SPSS Software 16 was used . The mean and standard deviation (SD) were calculated, since the data followed normal distribution. Independent t-test was used to demonstrate GTN of the two different groups. Moreover, in order to compare the qualitative variables, the Chi²- square test was used. A P-value<0.05 was considered as significant. 

## Results

One hundred two patients with a mean age of 27.7±7.37 were enrolled in the study. The youngest patient was 15 years old, while the eldest was 48years. Half of the patients (51; 50%) received MTX as the prophylactic drug, while the other half did not receive it. Liver function test of each patient is measured the same day that (s)he received the methotrexate four times per cycle. Finally, 23 patients (22.5 %) were developed with GTN, while 79 patients (77.5 %) were not. 

Among the patients with GTN, 12 (52.17%) had an increase of βHCG, 11 patients (47.82%) showed less than 10% decrease in βHCG (plateau). The results show that the first two inclusion criteria were seen mostly among the patients, and the last two were not met. Also, in the inclusion criteria, the first one was the most prevalent.

**Figure 1:**
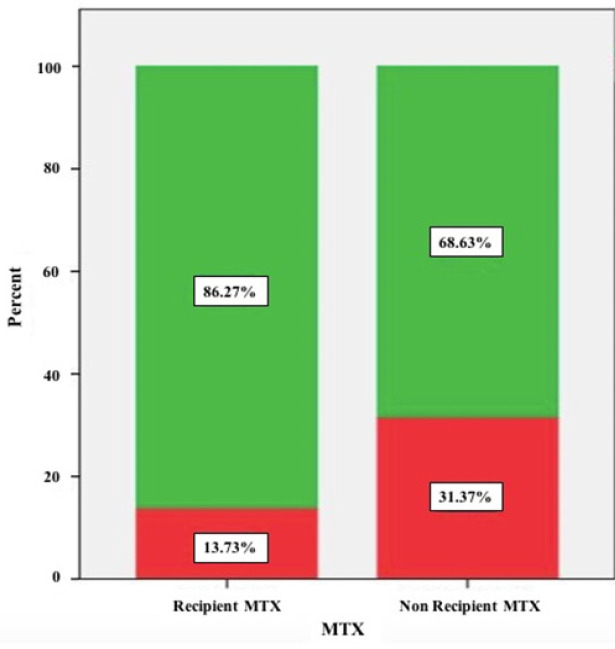
Description. Green color represents No GTN, and red color represents GTN

There was a significant difference between the number of patients with GTN in the recipient MTX group (7 women ~13.7 %) and the number of patients with GTN in the non-recipient MTX group (16 women ~ 31.4 %) with p-value =0.03, (Figure 1). There was a significant difference in average of the month to normalization of βHCG (spontaneous remission) between MTX recipient patients (2.5±1.33) and non-recipient MTX patients (3.2±1.21) (P<0.001). While, there was not any significant difference with regards to age (P=0.79), parity, gravidity average and the number of abortions in patients with GTN and those with no GTN (P>0.05). Pre-MTX receivers βHCG average in the two groups did not indicate any significant difference (P=0.31), neither did they show any significant difference after 48 h (P>0.05). 

After performing prophylactic chemotherapy, 12 patients who received MTX had AST above 40, and 14 patients had ALT above 40. In 6 of them, the liver enzymes were doubled at least. In such cases, the drug prescription was temporarily stopped. However, after normalization of liver enzymes, the chemotherapy was continuing until fulfilling taking four doses. 

## Discussion

Results of this study showed that prophylactic chemotherapy reduced the risk of GTN in women with high risk mole. The MTX receivers showed significantly more normal βHCG level (spontaneous remission) in a shorter time. This idea was started in a systematic review by Wang et al., (2017), which showed that the evidence to support this proposition is not significant, and needs further investigation. Additionally, various studies have indicated that prophylactic chemotherapy has no useful effect on low-risk molar pregnancies. Moreover, the GTN appearance in high risk cases has been reported to be about 50% (Gueye et al., 2014; Seckl, 2014). These two reasons encouraged the authors to consider only the high-risk cases. Moreover, a study that reviewed the meta-analysis results in low and high-risk pregnancies showed that the prophylactic chemotherapy could lower GTN in almost two third of the patients (Wang et al., 2017). The same results were obtained from another study, which only considered patients with high-risk molar pregnancies (Uberti et al., 2006).

Moreover, prophylactic chemotherapy usually performed in high-risk molar pregnancy patients with low accessibility to health care centers, and the patients (with the history of molar pregnancy evacuation) who are not coming back to have a follow up (Elza Maria Hartmann Uberti et al., 2009; Wang et al., 2017). In Latin America, about 44% of the patients won’t be followed up (Uberti et al., 2009). The country this study performed is a developing country, which attracts multiple international work force (immigrant). These kinds of patients are not interested to have a follow up due to the costs burden associated with it. So, this area is a bed stream for having such studies. 

Aminimoghaddam et al., (2017) concluded that the majority of patients affected by invasive mole had previous high-risk molar pregnancy. However, actinomycin D is a scientific substitute for MTX. A study in 2009, showed that there is no significant difference between the two drugs (Uberti et al., 2009). Uberti et al., (2009) had another approach and prescribed actinomycin D in patients with high-risk mole. Results showed that prophylactic chemotherapy could lower the GTN rate, as well as treatment costs, though the usage of this drug has no significant impact on the morbidity and diseases intensity (Vargas et al., 2014). This reduction was quantified in two studies; Uberti et al. showed that the use of actinomycin D prior to suction curettage reduces the GTN rate up to 46% (Uberti et al., 2006), while Cunha et al., (2009) found this reduction rate to be 76% (Uberti et al., 2009). However, this study practiced using MTX since actinomycin D was not available in the country this study performed. 

From a different perspective, some scholars proved the effectiveness of using leucovorin beside MTX (Aminimoghaddam et al., 2018; Wang et al., 2017; Yarandi et al., 2016), while this study had a focus on the level of toxicity, which makes it unique. A rise in liver enzyme was seen as a side effect of MTX, which engendered terminating MTX. This termination made the level of liver enzyme normal again. It should be said that this treatment was continued for a full treatment period as it was mentioned. 

## Future research

It is suggested to evaluate the risk of GTN by using prophylactic chemotherapy by conducting an RCT study. Additionally, drug resistance, toxicity, overall survival and reproductive outcome should be clarified in this study.
